# Supplementary material for: Content-rich biological network constructed by mining PubMed abstracts
Source: BMC Bioinformatics. 2004 Oct 8;5:147. doi: 10.1186/1471-2105-5-147 (PMC528731; doi:10.1186/1471-2105-5-147)
Supplement: Additional File 2 — The original results of the above study (non-essential files are deleted to keep the file size under the limit set by BMC bioinformatics). [file 1471-2105-5-147-S2.bz2 › chilibotAdditionalFile2/dip05/441ID9169459E10393/html/CCR3_CCL11.html]

 


 **CCR3** and **CCL11** 
  
Found 222 abstracts in PubMed, retrieved 05.  
 

 What does Google say? 
 PDF only 
| .edu only 

---

**Interactive relationship** (e.g. stimulation, inhibition, etc)

**Inhibitory relationship**- This migrational response to eotaxin  [ **CCL11** ]  was abrogated by anti  **CCR3**  monoclonal antibody and primed by preincubation with IL 5.  Ref: 12871220 ImmunologyImmunology,
**Neutral relationship**- An inhibitor of the eotaxin  [ **CCL11** ]   **CCR3**  binding interaction may have potential as an anti inflammatory drug for treatment of asthma, parasitic infections, and allergic disorders.  Ref: 12857386 J Biomol Screen, 2003
- Eotaxin  [ **CCL11** ] , an inducer of eosinophil migration and activation, exerts its activity by binding to  **CCR3** , the C C chemokine receptor 3  [ **CCR3** ] .  Ref: 12857386 J Biomol Screen, 2003
- Hence, eosinophil responses mediated by chemokines acting at  **CCR3**  may be regulated by two distinct mechanisms the antagonistic effects of CXCR3 ligands and the sequestration of  **CCL11**  by CXCR3 expressing cells.  Ref: 12884299 Eur J Immunol, 2003

**Non-interactive relationship** (e.g. studied together, co-existance, homology, etc.)

- An eosinophil active chemokine, eotaxin  [ **CCL11** ] , and its receptor, C C chemokine receptor 3  [ **CCR3** ] , are particularly attractive as novel targets of immunological intervention for the disease.  Ref: 12842759 Cytokine< MedlineTA>Cytokine, 2003
- To study mechanisms of progenitor cell mobilization in allergic reactions, we investigated fluctuations in the expression of the eotaxin  [ **CCL11** ]  receptor  [ **CCR3** ] , CC chemokine receptor 3  [ **CCR3** ]   **CCR3** , on CD34 cells from stable asthmatics following allergeni.e. antigen challenge.  Ref: 12871220 ImmunologyImmunology, 2003
- The examination of  **CCR3**  CCR1 chimeric constructs revealed that  **CCL11**  and CXCL11 share overlapping binding sites contained within the  **CCR3**  extracellular loops, a region that was previously shown to be essential for effective receptor activation.  Ref: 12884299 Eur J Immunol, 2003
- In contrast, the  **CCR3**  agonists  **CCL11** , CCL24 and CCL26 are involved in the recruitment of cells such as eosinophils and basophils during Th2 responses.  Ref: 12884299 Eur J Immunol, 2003
- Consistent with these results, infected cells from WT mice preferentially migrated in response to IP 10 CXCR3 ligand, while those from GKO mice migrated in response to eotaxin  [ **CCL11** ]   **CCL11**   **CCR3**  ligand.  Ref: 12875978 Am J Pathol, 2003
- A radioligand binding assay was developed using HEK cells transfected with  **CCR3** , with 125 I eotaxin  [ **CCL11** ]  as the ligand.  Ref: 12857386 J Biomol Screen, 2003
